# Evidence from phylogenetic and genome fingerprinting analyses suggests rapidly changing variation in *Halorubrum* and *Haloarcula* populations

**DOI:** 10.3389/fmicb.2014.00143

**Published:** 2014-04-09

**Authors:** Nikhil Ram Mohan, Matthew S. Fullmer, Andrea M. Makkay, Ryan Wheeler, Antonio Ventosa, Adit Naor, J. Peter Gogarten, R. Thane Papke

**Affiliations:** ^1^Department of Molecular and Cell Biology, University of ConnecticutStorrs, CT, USA; ^2^Department of Microbiology and Parasitology, University of SevilleSeville, Spain; ^3^Molecular Microbiology and Biotechnology, Tel Aviv UniversityTel Aviv, Israel

**Keywords:** Halobacteria, MLSA, genome fingerprinting, Aran-Bidgol lake, environmental population

## Abstract

Halobacteria require high NaCl concentrations for growth and are the dominant inhabitants of hypersaline environments above 15% NaCl. They are well-documented to be highly recombinogenic, both in frequency and in the range of exchange partners. In this study, we examine the genetic and genomic variation of cultured, naturally co-occurring environmental populations of Halobacteria. Sequence data from multiple loci (~2500 bp) identified many closely and more distantly related strains belonging to the genera *Halorubrum* and *Haloarcula*. Genome fingerprinting using a random priming PCR amplification method to analyze these isolates revealed diverse banding patterns across each of the genera and surprisingly even for isolates that are identical at the nucleotide level for five protein coding sequenced loci. This variance in genome structure even between identical multilocus sequence analysis (MLSA) haplotypes indicates that accumulation of genomic variation is rapid: faster than the rate of third codon substitutions.

## Introduction

Members of the class Halobacteria (Domain: Archaea; Phylum: Euryarchaeota) are the dominant inhabitants of hypersaline environments (Anton et al., 1999; Ghai et al., 2011). These hypersaline environments provide extreme growth conditions in the form of high salinity and ionic concentrations with variations in pH, and temperature (Oren, 2002). Such extreme conditions are necessary for Halobacteria, also called haloarchaea, to live. The environment is also subject to low solubility of gases, low diffusion rates, and very low water activity (Litchfield, 1998). To overcome many of these obstacles, haloarchaea can generate ATP from light energy (Lozier et al., 1975) and have gas vesicles to buoyantly lift themselves to the surface (Jones et al., 1991). Osmotic survival in these brines is managed by maintaining a cytosolic salinity in equilibrium with that of the environment, a feat that requires solubilized proteins under those conditions, and solved with a proteome enriched in acidic and depleted of basic amino acids (Oren, 2002).

Haloarchaea have a well-documented capacity for generating enormous amounts of genetic variation through horizontal gene transfer (HGT) (Papke et al., 2004, 2007; Cuadros-Orellana et al., 2007; Lynch et al., 2012; Naor et al., 2012; Williams et al., 2012; Demaere et al., 2013; Podell et al., 2013). From the very first genome sequence analysis of *Halobacterium* strain NRC-1, evidence was provided for the acquisition of aerobic respiration genes via HGT from Bacteria (Ng et al., 2000). Since then, several studies on specific genes of interest [e.g., rhodopsins (Sharma et al., 2007), ribosomal RNAs (Boucher et al., 2004), and tRNA synthetases (Andam et al., 2012)] have further demonstrated gene transfer into and among the haloarchaea. A recent report suggested that this process of generating diversity has been ongoing since before the group's last universal common ancestor and that HGT played a huge role in changing their physiology from an autotrophic anaerobe to a heterotrophic aerobe (Nelson-Sathi et al., 2012). Population genetics analysis on strains from the genus *Halorubrum* using multilocus sequence analysis (MLSA) demonstrated that alleles at different loci are unlinked indicating that homologous recombination (HR) is frequent enough within phylogenetically defined groups to randomize traits among individuals (Papke et al., 2004, 2007), an observation once considered unique to sexually reproducing eukaryotes. Analysis of 20 haloarchaeal genomes showed that there are no absolute barriers to HR, which occurs regularly and proportionally to genetic distance throughout the haloarchaea (Williams et al., 2012). Community analyses using metagenomics revealed that genes are coming and going quickly within *Haloquadratum walsbyi* populations, suggesting there may be very few identical genomes within the species (Legault et al., 2006; Cuadros-Orellana et al., 2007). Perhaps most striking is their ability to exchange large swaths of genetic information. Mating experiments between *Haloferax volcanii* and *Haloferax mediterranei* demonstrated between ~10 and 18% (~300–500 kb) of their chromosome could be transferred in a single fragment (Naor et al., 2012). Also, genomes of highly divergent strains (e.g., <75% average nucleotide identity) isolated from Deep Lake, Antarctica were shown to share many ~100% identical DNA sequences in fragments up to 35 Kb in length (Demaere et al., 2013).

MLSA has often been used as a technique for classifying microorganisms (Maiden et al., 1998), including halophiles (Papke et al., 2011; De la Haba et al., 2012), but it is also used to estimate population variation and gene flow (Feil et al., 2000). Assumptions using MLSA regarding how representative multiple genes are for capturing individual variation, and thus the appearance of clonality, can lead to erroneous conclusions. For instance, two strains may have identical sequences across multiple loci, but unexamined genomic variation might be high and belie the interpretation of little or no recombination. Indeed, studies are demonstrating that there are vast amounts of variation within bacterial species/populations. Environmental isolates with identical HSP-60 genes from a natural coastal *Vibrio* sp. population demonstrated that the overwhelming majority of individual strains were unique as determined by chromosome pulse field gel electrophoresis, with some strains differing by up to a megabase in genome size (Thompson et al., 2005). This variation in genome size and the existence of “open” (i.e., infinite) pan-genomes like that of *Prochlorococcus marinus* and others (Tettelin et al., 2008; Lapierre and Gogarten, 2009) suggest that HGT is so frequent that for at least some species every cell may be genetically distinct.

To get a better understanding for the genomic variation within closely related haloarchaeal strains we examined naturally co-occurring environmental strains from the genera *Halorubrum* and *Haloarcula* isolated from the Aran-Bidgol salt lake in Iran. We used MLSA to identify closely related strains, and a PCR genome fingerprinting technique that randomly primed amplification sites along the chromosome to generate a gel electrophoresis pattern that enabled us to inexpensively compare genomic variation of the isolates.

## Materials and methods

### Growth conditions and DNA extraction

Aran-Bidgol *Halorubrum* and *Haloarcula* spp. cultures were grown in Hv-YPC medium (Allers et al., 2004) at 37°C with agitation. DNA from haloarchaea was isolated as described in the Halohandbook (http://www.haloarchaea.com/resources/halohandbook/). Briefly, stationary-phase cells were pelleted at 10,000 ×*g*, supernatant was removed and the cells were lysed in distilled water. An equal volume of phenol was added, and the mixture was incubated at 65°C for 1 h prior to centrifugation to separate the phases. The aqueous phase was reserved and phenol extraction was repeated without incubation, and followed with a phenol/chloroform/iso-amyl alcohol (25:24:1) extraction. The DNA was precipitated with ethanol, washed, and resuspended in TE (10 mM tris, pH 8.0, 1 mM EDTA). Type strains were grown, and DNA was purified as described by Papke et al. (2011).

### Sequence acquisition for MLSA

Five housekeeping genes were amplified using PCR. The loci were *atpB*, *ef-2*, *glnA*, *ppsA*, and *rpoB* and the primers used for each locus are listed in Table 1. To more efficiently sequence PCR products, an 18 bp M13 sequencing primer was added to the 5′ end of each degenerate primer (Table 1). Each PCR reaction was 20 μl in volume. Phire Hot Start II DNA polymerase (Thermo Scientific) was used in the amplification reactions. The PCR reaction was run on a Mastercycler Ep Thermocycler (Eppendorf) using the following PCR cycle protocol: 30 s initial denaturation at 98°C, followed by 40 cycles of 30 s at 98°C, 5 s at the annealing temperature for each set of primers, and 15 s at 72°C. Final elongation occurred at 72°C for 1 min. Table 2 provides a detailed list of reagents and the PCR mixtures for each amplified locus. The PCR products were separated by gel electrophoresis with agarose (1%). Gels were stained with ethidium bromide. An exACTGene mid-range plus DNA ladder (Fisher Scientific International Inc.) was used to estimate the size of the amplicons, which were purified using Wizard SV gel and PCR cleanup system (Promega). The purified amplicons were sequenced by Genewiz Inc. The sequences obtained for the five genes in this study were submitted to Genbank under the following accession numbers: KJ152221–KJ152260, KJ152261–KJ152298, KJ152362–KJ152397, KJ152398–KJ152433, and KJ152323–KJ152361.

**Table 1:** **Degenerate primers used to PCR amplify and sequence the *atpB, ef-2, glnA, ppsA*, and *rpoB* genes for MLSA**.

**MLSA primer sequence 5′–3′**		
**Locus**	**Forward**	**Reverse**
atpB	tgt aaa acg acg gcc agt aac ggt gag scv ats aac cc	cag gaa aca gct atg act tca ggt cvg trt aca tgt a
ef-2	tgt aaa acg acg gcc agt atc cgc gct bta yaa stg g	cag gaa aca gct atg act ggt cga tgg wyt cga ahg g
glnA	tgt aaa acg acg gcc agt cag gta cgg gtt aca sga cgg	cag gaa aca gct atg acc ctc gcs ccg aar gac ctc gc
ppsA	tgt aaa acg acg gcc agt ccg cgg tar ccv agc atc gg	cag gaa aca gct atg aca tcg tca ccg acg arg gyg g
rpoB	tgt aaa acg acg gcc agt tcg aag agc cgg acg aca tgg	cag gaa aca gct atg acc ggt cag cac ctg bac cgg ncc

**Table 2:** **PCR conditions for each locus**.

	**atpB**	**ef-2**	**glnA**	**ppsA**	**rpoB**
Water (μl)	11.6	8.2	11.8	7.9	11.9
5× phire reaction buffer (μl)	4.0	4.0	4.0	4.0	4.0
DMSO (μl)	0.6	0	0.4	0.6	0.6
Acetamide (25%)	0	4.0	0	4.0	0
dNTP mix (10 mM)	0.4	0.4	0.4	0.4	0.4
Forward primer (10 mM)	1.0	1.0	1.0	1.0	1.0
Reverse primer (10 mM)	1.0	1.0	1.0	1.0	1.0
Phire hot start II DNA polymerase (μl)	0.4	0.4	0.4	0.4	0.4
Template DNA (20 ng μl^−1^)	1.0	1.0	1.0	0.7	0.7
Annealing temperature (°C)	60.0	61.0	69.6	66.0	63.7

### Phylogenetic analysis

Type strain genomes were obtained from the NCBI ftp repository. Blast searches identified DNA top hits for each MLSA target gene (*atpB*, *ef-2*, *glnA*, *ppsA*, and *rpoB*) in each genome. Multiple-sequence alignments (MSAs) were created from the DNA genome hits as well as the PCR amplicons using MUSCLE (Edgar, 2004) (alignments available upon request) with its refine function. The MSA length was manually trimmed down to the lengths of the PCR amplicons. In-house scripts created a concatenated alignment of all five genes. A model of evolution was determined using the Akaike Information Criterion with correction for small sample size (AICc). The jModelTest 2.1.4 (Darriba et al., 2012) program was used to compute likelihoods from the nucleotide alignment and to perform the AICc test (Akaike, 1974). The AICc reported the best-fitting model to be GTR + Gamma estimation + Invariable site estimation. A maximum likelihood (ML) phylogeny was generated from the concatenated MSA using the PhyML v3.0_360–500 (Guindon et al., 2010). The model used in PhyML corresponded to the one favored by jModeltest: GTR model, estimated p-invar, 4 substitution rate categories, estimated gamma distribution with 100 bootstrap replicates. The number of nucleotide differences in pairwise comparisons were determined using MEGA 5 (Tamura et al., 2011).

### Genomic fingerprinting

In total, DNA from 81 haloarchaeal type strains and 43 isolates from the Aran-Bidgol lake were tested. Each primer selected has successfully been used in genome fingerprinting in previous studies. Primers P1 and P2 were used to fingerprint *Vibrio harveyi* bacteriophages (Shivu et al., 2007), primers OPA-9 and OPA-13 were used to asses marine viral richness (Winget and Wommack, 2008). The last primer, FALL-A was adapted from the primer used (Barrangou et al., 2002; Winget and Wommack, 2008) to study bacteriophages isolated from an industrial sauerkraut fermentation. Amplification conditions for each strain were equal to enable accurate comparison between banding patterns obtained. Each sample was diluted to 20 ng μl^−1^ and amplified within the following reaction mixture: 12.5 μl SYBR Universal Faststart Mastermix (Roche), 4.5 μl dH_2_0, 1.5 μl for each of five primers at 10 ng μl^−1^ (see Table 3), and 0.5 μl of template DNA. Two thermocycler programs were used in succession. The first included an initial 10 min denaturation at 94°C, followed by 4 cycles of a 45 s denaturation also at 94°C, annealing at 30°C for 2 min, and extension at 72°C for 50 s. This was followed by another 35 cycle program: 94°C for 17 s, 36°C for 30 s, and 72°C for 45 s, and a final extension for 10 min at 72°C. The aim of these repeated programs with low annealing temperatures and long annealing times is to produce as many non-specific bands as possible for each sample, increasing the resolving power of the method. Strains were amplified in triplicate to ensure that a repeatable banding pattern could be obtained.

**Table 3:** **Random primers for genomic fingerprinting**.

**Primers**	
**Primer name**	**Sequence**
P1	5′-CCGCAGCCAA-3′
P2	5′-ACGGGCAGC-3′
OPA-9	5′-GGGTAACGCC-3′
OPA-13	5′-CAGCAGCCAC-3′
FALL-A	5′-ACGCGCCCTG-3′

### Gel electrophoresis

Reactions mixtures from PCR experiments were held at 4°C prior to electrophoresis. Standard DNA electrophoresis was carried out with replicates from each strain. Gels were 1.5% agarose and run at 12 v for 16 h at 4°C with the goal of producing crisp bands easily distinguishable by the analysis software. Gels were stained with ethidium bromide prior to imaging.

### Imaging and analysis

A digital image of each gel was created using a GelDoc (UVP). Images were then analyzed using the Phoretix 1D Pro program from the TotalLab Inc. (www.totallab.com). Banding patterns were standardized for cross gel comparisons by calibrating Rf lines on individual gels. Phoretix 1D Pro converts banding patterns into a format that can be used to produce a dendrogram comparing the differences and similarities between the patterns of amplicons. The final dendrogram was created within Phoretix 1D Pro using UPGMA statistical analysis on Dice coefficients (Dice, 1945) for each of the lanes. A measure of the correlation between the matrix similarities and the dendrogram derived similarities, the cophenetic correlation coefficients (Sokal and Rohlf, 1962) were determined for each sub-cluster of the dendrogram and displayed on the nodes of the constructed dendrograms to estimate the robustness of each cluster.

## Results

### Genomic fingerprinting

**Figure 1:**
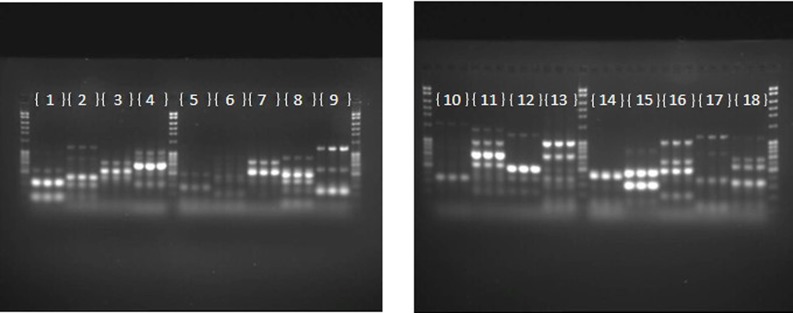
**Repeatability of the fingerprinting technique.** Each number represents a type strain analyzed in triplicate. (1) *Halorubrum arcis* JCM 13916 (2) *Halorubrum coriense* DSM 10284 (3) *Halorubrum distributum* JCM 9100 (4) *Halorubrum ejinorense* JCM 14265 (5) *Halorubrum lacusprofundi* ATCC 49239 (6) *Halorubrum lipolyticum* DSM 21995 (7) *Halorubrum litoreum* JCM 13561 (8) *Halorubrum saccharovorum* DSM 1137 (9) *Halorubrum sodomense* JCM 8880 (10) *Halorubrum tebenquichense* DSM 14210 (11) *Halorubrum terrestre* JCM 10247 (12) *Halorubrum tibetense* JCM 11889 (13) *Halorubrum trapanicum* JCM 10477 (14) *Halorubrum vacuolatum* JCM 9060 (15) *Halorubrum xinjiangense* JCM 12388 (16) *Halosarcina pallida* JCM 14848 (17) *Halosimplex carlsbadense* JCM 11222 (18) *Halostagnicola larsenii* JCM 13463.

The repeatability of banding patterns, and thus the success of the fingerprinting technique was tested on 81 haloarchaeal type strains. The PCR on each of the 81 strains was run in triplicate and the products were run on adjacent wells. Figure 1 demonstrates results of the banding pattern for 18 out of the 81 type strains, 15 from the genus *Halorubrum*, and one each from the genus *Halosarcina, Halosimplex*, and *Halostagnicola.* Repeatability for the other 63 was examined and they were consistent, as in Figure 1 (data are not shown). Repeatability of the technique indicated robustness of the conditions and primers used and provide confidence for estimating variation between strains.

**Figure 2:**
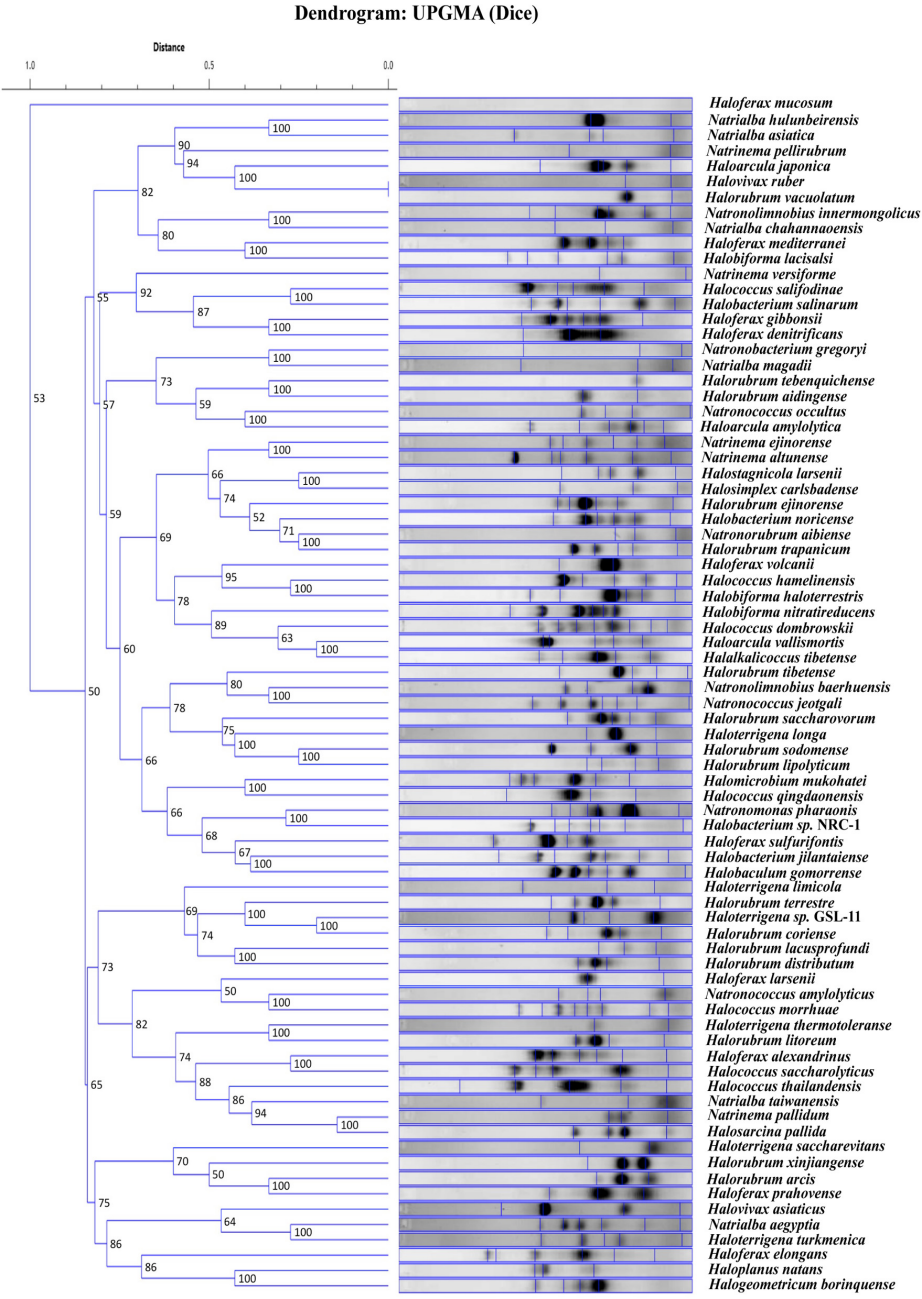
**UPGMA dendrogram comparing banding patterns between type strains.** The numbers displayed at the nodes represent the cophenetic correlation coefficients.

We were interested to know if the random primers can be used as a screening technique. If banding patterns could reliably demonstrate similarity within genera for instance, newly cultured yet unidentified strains could be easily screened and a general taxonomic decision could be made. Therefore, the banding patterns for the 81 total haloarchaeal type strains were assessed using software that produced a dendrogram of the genomic fingerprints. Figure 2 is the UPGMA dendrogram determined for the above type strains. Compared to other studies (e.g., Shivu et al., 2007; Winget and Wommack, 2008), our genome fingerprinting technique offers very little banding pattern complexity. There are two possible reasons—the primers were designed for systems other than the haloarchaea and adopted for our purposes, and PCR bias, though if it occurs is reproducible (see Figure 1). Yet, species specific banding patterns observed earlier in haloarchaea (Martinez-Murcia and Rodriguez-Valera, 1994) are also observed here; each species appears to have a unique banding pattern. However, there is very little clustering at the genus level. For instance, some species within the same genus have similar banding patterns according to the dendrogram analysis (e.g., *Natrinema ejinorense* and *Natrinema altunense*) but other species from the same genus are found elsewhere (e.g., *Natrinema pelliruberum* and *Natrinema versiforme*). This pattern is observed for all the genera for which several species were analyzed (e.g., *Halorubrum*, *Haloferax*). Thus, this DNA fingerprinting should not be used to classify isolates to a genus level. The observed amount of variation displayed among species within the same genus, led to the hypothesis that this technique might also detect genomic variation among strains within the same species. Therefore, we tested this fingerprinting technique on several populations of naturally co-occurring closely and distantly related strains.

### MLSA on environmental strains

**Figure 3:**
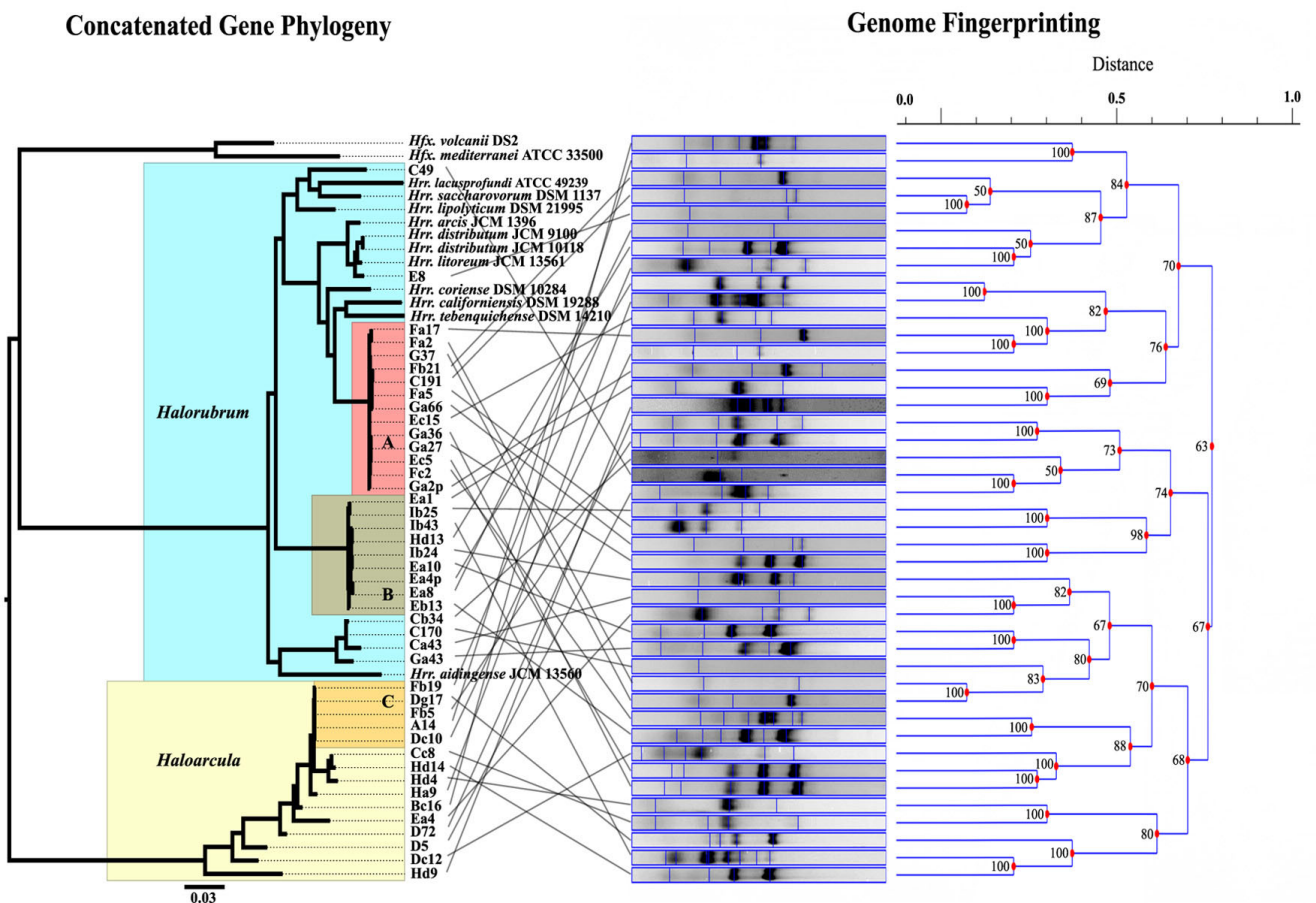
**Comparison of the maximum likelihood tree computed from the concatenation of five housekeeping genes, and the UPGMA dendrogram determined from the banding patterns of the genome fingerprinting.** Lines between tree and dendrogram connect the same strain in the different analyses.

MLSA was performed in order to determine the genetic variation, and the evolutionary relationships of the isolates from Aran-Bidgol lake. Multiple sequence alignments were constructed from individual locus data from the new isolates and from genome data deposited in the NCBI database of type strains. Concatenated alignments were made from these and then a phylogenetic tree was constructed. The Aran-Bidgol isolates clustered into two main genera; *Halorubrum* and *Haloarcula* (Figure 3)*.* Two polytomous groups, A and B, were observed within the genus *Halorubrum* and depicts evidence for distinct phylogroups with low sequence diversity as first seen for Spanish and Algerian isolates (Papke et al., 2007). Pairwise comparison of the number of nucleotides different within each of these phylogroups was carried out using MEGA 5 (Tamura et al., 2011). In both groups A and B, no two isolates had more than 10 nucleotide differences from one another across the concatenation of ~2500 bp (i.e., <1% sequence divergence; Table 4). This also holds true for group C (Table 5) within the *Haloarcula* cluster.

**Table 4:** **Pairwise comparison of number of nucleotide differences within polytomous Groups A and B defined on the maximum likelihood tree**.

GROUP A	Ga27					0	7	8	5	7	10	9	6	Ea8	GROUP B
	Ec5	5					7	8	5	7	10	9	6	Ea4p	
	Ec15	8	5					5	2	8	7	6	5	Ea10	
	Ga66	7	8	7					5	9	8	7	4	Hd13	
	Fc2	5	4	5	6					6	9	8	3	Ib24	
	Fa2	1	1	1	0	1					5	4	7	Eb13	
	Fa5	7	8	7	4	6	0					1	8	Ib25	
	Fa17	2	2	2	0	2	0	1					7	Ea1	
	C191	8	9	8	5	7	0	1	1					Ib43	
	Fb21	8	9	8	5	7	0	1	1	0					
	Ga36	4	3	6	7	3	1	7	3	8	8				
	G37	8	3	4	7	5	0	5	1	6	6	6			
	Ga2p	6	5	4	5	3	0	5	1	6	6	4	4		

**Table 5:**
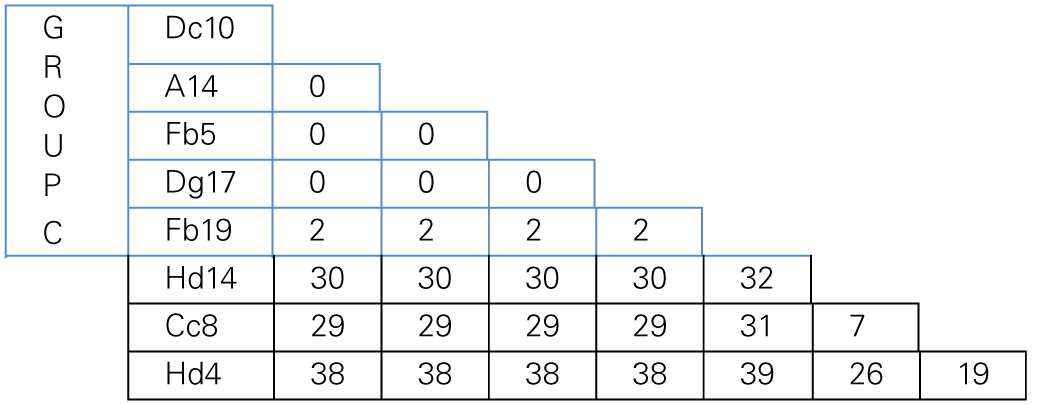
**Pairwise comparison of number of nucleotide differences within polytomous Group C defined on the maximum likelihood tree**.

Cells in blue represent members of Group C and cells in black represent the neighboring cluster on the ML tree.

### Fingerprinting the Aran-Bidgol strains

Genomic fingerprint analysis was run on each of the Aran-Bidgol lake environmental isolates. Banding patterns for each individual were generated and compared for similarity by dendrogram construction. The fingerprints and resulting dendrogram were then compared to the ML tree constructed from the MLSA data (Figure 3) for relating genetic and genomic variation within populations. It is noteworthy that despite limited numbers of bands produced for fingerprinting analysis, closely related strains from a single phylogroup displayed numerous variations in banding patterns, many of which were dissimilar to each other as determined by the dendrogram analysis. These widely different banding patterns reflect the variation in individual genomes. Comparison between sequence and banding pattern similarity demonstrates a lot of variation and no discernable patterns of relatedness even between strains that have zero differences across ~2500 nucleotides. Banding patterns of isolates within the genus *Halorubrum* seem as different as the banding patterns of isolates between the genera *Halorubrum* and *Haloarcula.* In some cases identical MLSA haplotypes have identical fingerprint patterns. We believe this can be attributed to the relatively low complexity of fingerprint bands produced, rather than two strains having identical genomes, and in such cases other methods of comparison like genome sequencing might reveal additional differences.

## Discussion

Our study employed DNA sequencing of multiple protein coding loci and random genomic amplification to test for variation in haloarchaeal isolates cultivated from the same location under the same conditions. The concatenated ML tree in Figure 3, and the number of pairwise nucleotide polymorphisms in Tables 4, 5, show that many isolates are closely related to one another across the five loci and are more or less indistinguishable from each other by these methods. However, the DNA fingerprinting analysis on these same isolates revealed additional variation not captured by MLSA, indicating genomic changes occur faster than the rate of substitution in redundant codon positions. Unfortunately, the deeper branches of the UPGMA hierarchical clustering dendrogram are unreliable for determining relationships and do not provide a good description of the measured Dice coefficients. Yet, shallower branches in the clustering diagram that are a good representation of the banding pattern differences show conflict with the MLSA phylogeny (Figure 3). Though the fingerprinting technique did not yield patterns of relatedness at the species level or genus level, it did demonstrate the high probability that the genomes of each isolates are unique. Whether that uniqueness is based on gene content or in genomic arrangements is undeterminable from this analysis.

However, given the known propensity for HGT in Halobacteria (Papke et al., 2004, 2007; Cuadros-Orellana et al., 2007; Lynch et al., 2012; Naor et al., 2012; Williams et al., 2012; Demaere et al., 2013; Podell et al., 2013), we surmise the fingerprint banding-pattern differences are largely due to gene transfer events. Discovery of recombinant hybrids (Naor et al., 2012) and the identification of enormous identical segments shared among the genomes of phylogenetically distant genera (Demaere et al., 2013) indicates the haloarchaea are subject to immense genomic variability from single gene transfer events. In another study, an influx of 303 transferred genes into *Haloferax mucosum* and *Haloferax mediterranei* were mostly of unknown function with some known transporters (Lynch et al., 2012), which is similar to the types of genes observed in the highly recombinogenic genomic islands of *Haloquadratum waslbyi* (Cuadros-Orellana et al., 2007). The *H. waslbyi* genome is 47.9% GC, but its genomic islands are GC rich by comparison, and enriched in transposable and repeat elements (Bolhuis et al., 2006) indicating a role for viruses in generating genomic diversity (Cuadros-Orellana et al., 2007). Similar to *H. walsbyi*, the genome of *Halobacterium* NRC-1 was interspersed with 91 insertion sequence elements of diverse GC compositions (Ng et al., 2000; Kennedy et al., 2001). Apart from HR, IS elements have been attributed to inactivating the bacterio-opsin gene in *Halobacterium halobium* (Dassarma et al., 1983) and causing genomic rearrangements at AT-rich regions in *Halobacterium* NRC-1 (Kennedy et al., 2001). Moreover, recent analysis indicates these Aran-Bidgol lake isolates display enormous variation in whole genome content with differences in group A ranging from 0.01 up to 0.51 Mb and from 0.07 up to 0.30 Mb in group B (Fullmer et al., 2014). Therefore, we hypothesize the drastic differences in fingerprints observed for the closest relatives (e.g., strains from groups A, B, and C) are more likely due to HGT, possibly mediated by insertion sequence elements (Dassarma et al., 1983; Ng et al., 2000; Kennedy et al., 2001), tRNAs (Naor et al., 2012), or other factors, rather than genome rearrangements.

We further suggest that the fingerprint banding patterns, especially for those within groups A, B, and C, were unlikely due to mutational events. Haloarchaea have low rates of spontaneous mutation, having been measured at 1.90 × 10^−8^ mutational events per cell division (Mackwan et al., 2007). Furthermore, haloarchaea are considered to have a high capacity for repairing DNA, as they have demonstrated the ability to survive radiation and desiccation damaged DNA (McCready, 1996; Kottemann et al., 2005), which is probably due to the prevalence of polyploidy through the process of gene conversion (Lange et al., 2011). Preliminary *in silico* analysis to determine the binding sites for each of the five primers in *Haloquadratum walsbyi* DSM 16790 and *Halorubrum lacusprofundi* revealed priming mostly in conserved loci, although a few phage related loci were also detected. Because many of the compared strains are very closely related, having only a few (or zero) nucleotide polymorphisms in the ~2500 sequenced base pairs, yet display enormous differences in fingerprint banding patterns, it would be unlikely that a few, or even one of the PCR binding sites in every strain within groups A, B, or C, would be mutated. Therefore, substitutions in PCR primer binding sites seem unlikely to have played a role in generating all the observed differences in banding patterns, especially those from closely related strains.

Analysis of five housekeeping genes demonstrates the isolates form genetically similar and distinct populations in a single environmental community and yet each genome is apparently different. This observation agrees well with expectations from the distributed genome hypothesis (Ehrlich et al., 2010). According to this, the non-core genes available in the pangenome pool are dispensed uniquely amongst the individual cells of a species. The differences in haloarchaeal genomic banding patterns suggests that in nature populations are made of highly varied individuals rather than clones of a single individual. The number of distinct genotypes observed, most likely due to gene flow, suggests that haloarchaeal cells are acquiring genomic variation within populations at a rate faster than redundant codon position substitutions, and possibly at every replication event. Distribution of the non-core genes within a highly recombining population (defined by MLSA phylogeny) theoretically enables the individual to quickly adapt to new environmental selection conditions, especially virus predation (Cuadros-Orellana et al., 2007) but may also result from random processes like neutral drift (Gogarten and Townsend, 2005).

## Author contributions

R. Thane Papke, J. Peter Gogarten, and Antonio Ventosa conceived the researched. Nikhil Ram Mohan, Matthew S. Fullmer, Andrea M. Makkay, and Ryan Wheeler gathered data, and performed the analyses. Nikhil Ram Mohan, Matthew S. Fullmer, Andrea M. Makkay, Ryan Wheeler, Antonio Ventosa, J. Peter Gogarten, and R. Thane Papke wrote the manuscript.

### Conflict of interest statement

The authors declare that the research was conducted in the absence of any commercial or financial relationships that could be construed as a potential conflict of interest.
